# Effort-reward imbalance and perceived quality of patient care: a cross-sectional study among physicians in Germany

**DOI:** 10.1186/s12889-016-3016-y

**Published:** 2016-04-18

**Authors:** Adrian Loerbroks, Matthias Weigl, Jian Li, Peter Angerer

**Affiliations:** Institute of Occupational and Social Medicine, Centre for Health and Society, Faculty of Medicine, University of Düsseldorf, Universitätsstraße 1, 40225 Düsseldorf, Germany; Institute and Outpatient Clinic for Occupational, Social, and Environmental Medicine, Ludwig-Maximilians-University, Ziemssenstrasse 1, 80336 Munich, Germany

**Keywords:** Effort-reward imbalance, Health services, Physicians, Quality of care, Work stress

## Abstract

**Background:**

Work stress may impair physicians’ ability to provide high quality patient care. Prior research remains however sparse and has insufficiently explored explanations for this relationship. It has been suggested that physicians’ poor mental health is one potential explanatory factor. We drew on a well-established model to measure work stress (the effort-reward imbalance [ERI] model) in order to test this hypothesis. Further, to address another research gap and to potentially inform the development of better-targeted interventions, we aimed to examine associations of individual ERI constructs with the quality of care.

**Methods:**

We used cross-sectional data, which had been collected in 2014 among 416 physicians in Germany. ERI constructs (i.e. effort, reward, the ERI ratio, and overcommitment) were measured by the established 23-item questionnaire. Physicians’ perceptions of quality of care were assessed by a six-item instrument inquiring after poor care practices or attitudes. Physicians’ mental health was operationalized by the state scale of the Spielberger's State-Trait Depression Scales. We used both continuous and categorized dependent and independent variables in multivariable linear and logistic regression analyses.

**Results:**

Both an increasing ERI ratio and increasing effort were associated with poorer quality of care while increasing rewards were related to better care. Physicians’ depressive symptoms did not affect these associations substantially. Associations with overcommitment were weak and attenuated to non-significant levels by correction for depressive symptoms. The level of overcommitment did not modify associations between the ERI ratio and quality of care.

**Conclusions:**

Our study suggests that high work-related efforts and low rewards are associated with reports of poorer patient care among physicians, irrespectively of physicians’ depressive symptoms. Quality of patient care may thus be improved by concurrently reducing effort and increasing rewards among physicians.

## Background

Abundant research has documented that work stress is associated with an increased risk of poor mental and physical health outcomes among employees [1–5]. This relationship has been established for various professions, including physicians [6, 7], who are exposed to particularly high work stress [8]. Importantly though, work stress may not only predict poor health among physicians, but may also compromise their ability to provide care thereby contributing to poorer service quality and worse patient health [9, 10].

A recent review on the most prominent work stress models characterizing physicians’ work conditions classified three major indicators of quality of care [9], these were, 1) medical errors, e.g. related to the dosing of medication; 2) successful treatment of patients and absence of complications; and 3) physician or patient perceptions of the quality of care. Each of these indicators provides unique information on relevant care outcomes: medical errors relate to the safety of care while successful treatment reflects its effectiveness [9]. Patients’ and physicians’ perceptions of care are important and robust indicators of care quality and relate to patient-centeredness [9] and patient health [11–13].

Insights into the relationships of work stress and the perceived quality of care in physicians remain however sparse and inconclusive [9]. To our knowledge, only two studies drew on physicians’ evaluations and experiences [14, 15]. The more recent of those prior studies was experimental [14]. That study tested, and confirmed, that a workplace intervention effectively reduced the frequency of workflow interruptions among hospital pediatricians and improved the self-reported quality of care. The other previous study, by Klein et al. [15], was a cross-sectional survey which drew on the best-established theoretical models to assess work stress; these were, first, the demand-control model [16] which emphasizes the co-occurrence of high demands and lack of control as key contributor to work stress, and second, the effort-reward imbalance model (ERI) [17], whose theoretical crux is that efforts at work exceed the received rewards (the latter being salary, recognition, job security and promotion). An additional component of the ERI model is overcommitment, which reflects a personality trait associated with an excessive and intrinsically motivated work drive [18]. According to the ERI model, overcommitment is not only considered to operate as an independent predictor of poor outcomes, but is also hypothesized to amplify the adverse effects of effort-to-reward imbalance [19]. In the study by Klein et al. [15], work stress according to the demand-control model showed fairly consistent associations with different measures of physician-perceived quality of care. The results for the ERI model were however less consistent with the strength of associations varying across the employed care outcome measures.

While those prior studies provided valuable insights, there are important and unaddressed limitations and research gaps: Firstly, it has been hypothesized that poor mental health may mediate potential links between work stress and poor patient care [9, 10]. This notion is consistent with prospective studies documenting that work stress predicts poor mental health [1] which in turn is predictive of worse health care delivery [20–22]. Regrettably though physicians’ mental health has rarely been considered as an explanatory or mediating factor in prior research [9]. Alternatively to its potential mediating role, physicians’ mental health may also explain any observed association if it is conceptualized as a confounder or as a proxy measure correcting for participants’ affect-related tendencies for systematic over-reporting (i.e., common-method variance inducing spurious associations). To increase the validity of estimations, it is thus of interest to report associations between work stress and quality of care without and with correction for health care providers’ mental health.

The second research gap pertains to the observation that prior research has failed to examine the ERI model in all its facets [17]. Klein et al. [15] considered the ERI ratio as a determinant of patient care, but did not examine the individual effort and reward components separately. While the ERI ratio indisputably represents the key construct of the ERI model, it is nevertheless of interest to examine the individual contributions of effort and reward. For instance, the mechanisms linking these constructs to poor patient care may differ between efforts (e.g. errors and lack of empathy due to cognitive and emotional depletion by high efforts) and reward (e.g. frustration and lack of motivation due to low rewards). Moreover, each component calls for specifically tailored intervention strategies: if effort was the main determinant, quality of care is to be improved by effectively reducing physicians’ work demands and workload, e.g., by increasing staffing. By contrast, if low rewards were more important for poor patient care, work-related rewards needed to be fostered, e.g., by increasing salaries or fairer allocation arrangements, by transparent communication of career prospects or addressing of the organizational climate to increase perceptions of support and recognition. Furthermore, as stated above, the ERI model asserts that any link between effort-to-reward imbalance and health is more pronounced in those with high overcommitment [19]. Notably, this hypothesis has been expanded to and confirmed for job performance: in a study of company workers, for instance, overcommitment had been found to aggravate links between ERI and job performance [23]. While it is of interest to also examine the potential ERI-overcommitment interaction related to physician-perceived quality of care, this gap has not yet been addressed.

A third limitation of the existent evidence on work stress and physician-perceived quality of care is that earlier studies were restricted to physicians in hospitals and to single medical specialities, i.e., surgery [15] or pediatrics [14]. The nature of psychosocial work environments varies considerably between hospital and primary care physicians [8, 24]. Further, it seems plausible that the quality of care practices and their implications are (partly) determined by the work setting (e.g. inpatient versus outpatient care), and through care procedures, which vary considerably across medical specialties. The current knowledge base thus suffers from limited generalizability beyond hospital settings and beyond the researched medical specialties.

We set out to examine associations between physicians’ work stress, in terms of the ERI model, and physician-reported quality of care while addressing the above-mentioned research gaps. Our objectives were therefore 1) to present multivariable estimates with and without adjustment for physicians’ mental health, and 2) to fully examine potential associations of the individual ERI constructs with quality of care. Furthermore, the current study was based on a well-characterized sample of physicians from various medical specialties working in various health care sectors (i.e., hospitals and primary care).

## Methods

### Study population

In 2004, we initiated a cohort study on work conditions and health among junior physicians (the Munich Physician Study) [6, 25, 26]. Briefly, we identified a random sample of 1000 physicians, who were a) in their second or third year of medical residency and b) worked in hospitals in the city of Munich or surrounding communities. A total of 621 physicians provided informed consent and returned their questionnaire. Respondents were followed-up in 2005 (*n* = 561, 90.3 % of the baseline sample), in 2007 (*n* = 507, 81.6 %), and in 2014 (*n* = 450, 72.5 %). While questionnaires at each assessment covered, among others, work conditions, educational issues, career prospects, lifestyles and health, information on the perceived quality of care was only gathered in 2014. The present analyses were therefore limited to the cross-sectional 2014 sample. We further restricted this sample to those who reported to currently work in medical care (*n* = 416) as opposed to public health, health insurances, research or administration. Our study was approved by the Institutional Review Board of the Medical Faculty of the Ludwig-Maximilians-University Munich, Germany. All respondents provided informed consent prior to their participation in our study. Additional details on the Munich Physician Study can be found elsewhere [6].

### Study context

The majority of German hospitals are run in public or non-profit ownership. After graduation from university, early-career physicians in Germany start their post-graduate specialty training in hospitals for a period of about 5 years. After completion of all requirements they either continue to work as specialists in hospitals or medical centers or start their own private practice, mostly in a self-employed manner. Increasing attention for hospital physicians’ work life arises from increasing economic pressure on hospitals and stricter EU working time regulations both acting in concert with shortage of physician staff due to sociodemographic changes and alternative career choices.

### Questionnaire

#### Effort-reward imbalance

Effort-reward imbalance was assessed by the established 23-item questionnaire [17]. This instrument measures effort by six items, reward by 11 items and overcommitment by six items. Responses are provided on a 4-point Likert scale inquiring participants to endorse their level of agreement ranging from “I strongly disagree” (=1) to “I strongly agree” (=4). We calculated scores for effort, reward and overcommitment by adding the respective item scores. Consequently, the potential range of scores is 6 to 24 for both the effort and the overcommitment scale and 11 to 44 for the reward scale. Higher scores indicate higher levels of the respective dimension. In line with the usual analytical strategy of the ERI [15, 25], we further calculated the ratio between effort and reward (weighted by the number of items), which operationalizes the key theoretical assumption of ERI model. A ratio score exceeding 1.0 is considered to reflect effort-to-reward-imbalance [17, 18]. In our study, Cronbach’s alphas for the effort, reward, and overcommitment scale were sufficient equaling 0.79, 0.87, and 0.79, respectively.

#### Quality of care

Perceived quality of care was measured by an 8-item questionnaire developed in a study among physicians in the US [27]. The items are presented as statements and cover adverse care practices and deficient care attitudes. Five items capture poor care practices such as incomplete discussion of treatment options with patients, omission of diagnostic tests, and discharge of patients to reduce the workload. Three items cover deficient care attitudes, such as little emotional reaction to a patient’s death or feelings of guilt because of disrespecting interactions with a patient. Physicians were asked to report frequencies on a 5-point Likert scale with response options from “never”, “once”, “several times per year”, “monthly” to “weekly”. We translated the English-language items to German and performed an independent back-translation. Inconsistencies were discussed in the study team until consensus was reached. As acknowledged by the developers of the original version, the questionnaire and its potential subscales have not yet been validated [27]. We therefore explored the psychometric properties of our German questionnaire. First, we ran exploratory factor analyses (EFA) to identify potential subscales using orthogonal rotation (Varimax). The number of potential factors was deduced from the inspection of the scree plot and based on Kaiser’s criterion [28], i.e. dropping all components with Eigenvalues <1.0. EFAs suggested that all items clustered into one single factor. We then inspected the factor loadings by each item considering loadings >0.5 as meaningful [29]. Two items did not meet this threshold and were excluded. One of these items covered how frequently a respondent had ordered restraints or medication for an agitated patient without evaluating him or her. The second excluded item addressed how frequently a respondent had little emotional reaction to the death of one of his/her patients [27]. We then re-ran the EFA based on the remaining six items. Again, a uni-factorial structure was suggested with factor loadings of ≥0.69 for each item. The items showed good internal consistently (Cronbach’s alpha = 0.82). For statistical analyses, we constructed a mean score with higher scores reflecting poorer care (potential score range = 6–30).

#### Poor mental health

We operationalized poor mental health by the 10-item state scale of German Spielberger's State-Trait Depression Scales [26, 30]. This scale assesses the current experience of characteristic cognitive and affective symptoms of depression. Respondents are asked to endorse their level of agreement on a 4-point Likert scale, which translates into a mean score with a potential range from 1 (low depressive symptoms) to 4 (high).

### Statistical analyses

Owing to the limited size of our sample, our primary statistical analysis was based on a continuous dependent variable (i.e. quality of care) and continuous independent variables (i.e. ERI construct z-scores). This approach was adopted because it provides more statistical power than analyses of categorized variables. Using linear regression modeling we examined the associations of each ERI construct with quality of care while testing for mediation by depressive symptoms. Associations were adjusted for age, sex, the working environment (i.e. working in inpatient versus outpatient care) and the occupational standing (leadership position versus no leadership position) [15]. In line with recommended approaches to mediation analyses [31], we examined 1) whether a given ERI construct was associated with depressive symptoms, 2) whether depressive symptoms were related to quality of care, 3) whether ERI constructs were linked to quality of care, and 4) to what extent the latter relationship was attenuated by additional adjustment for depressive symptoms. The size of the indirect effect and bias-corrected 95 % confidence intervals (CIs) were obtained through bootstrap techniques with 1000 replications [32].

We conducted further analyses (i.e. secondary analyses) to render the results from our study comparable to those from earlier work [15], that is, logistic regression analyses to estimate odds ratios (ORs) and corresponding 95 % CIs. In those analyses, the effort-reward ratio was dichotomized by its theoretical cut-off (i.e. >1.0 versus ≤1.0). As cut-offs have neither been established for effort, reward, or overcommitment nor for our quality of care outcome, we dichotomized these variables based on the respective tertile into high (top tertile) versus low (remaining tertiles) in keeping with previous research [15]. In addition, we employed continuous ERI variables (z-scores). We first ran unadjusted models. Second, as mentioned above, we controlled for the potential confounding effects of age, sex, and the working environment in keeping with prior studies [15]. In a final step, these models were additionally adjusted for depressive symptoms. The fit of the logistic regression models was assessed and confirmed using the Hosmer-Lemeshow test (i.e., *p*-values >0.05).

Finally, we examined the interaction of ERI and overcommitment related to quality of care. To maximize the statistical power for such interaction tests we employed continuous variables in linear regression models. We ran adjusted models (both with and without depression), which included the ERI variable, the overcommitment variable as well as the ERI x overcommitment interaction term.

## Results

On average, participants were in their early 40s, about half was female, and half worked in a leadership position (Table 1). Our sample displayed large occupational diversity with physicians from a broad range of medical specialties as well as physicians working in both in-patient care (41.1 %) and outpatient care settings (58.9 %). Bearing in mind the potential score ranges, depressive symptoms levels were rather low and overcommitment was, on average, at intermediate levels. Efforts and rewards were fairly high. The mean ERI score was 1.1 (standard deviation [SD] = 0.4) thereby exceeding the cut-off of 1.0 for effort-reward imbalance. When this cut-off was applied, we found that 57 % of the participating physicians were exposed to high work stress in terms of the ERI model.

**Table 1:** Characteristics of the study population (*n* = 416)

Characteristic		
Age in years, mean (SD)		40.1 (2.9)
Sex, *n* (%)	Men	205 (49.3)
	Women	211 (50.7)
Leadership position, *n* (%)	Yes	197 (51.0)
	No	189 (49.0)
Working environment, *n* (%)	In-patient care	166 (41.1)
	Out-patient care	238 (58.9)
Most frequent medical specialties^a^, *n* (%)	Internal medicine	96 (23.2)
	General medicine	55 (13.3)
	Anesthesia	48 (11.6)
	Surgery	33 (8.0)
	Pediatrics	31 (7.5)
	Gynecology	24 (5.8)
Depression score, mean (SD)		1.8 (0.5)
Effort score, mean (SD)		17.9 (3.5)
Reward score, mean (SD)		31.2 (5.8)
ERI score, mean (SD)		1.1 (0.4)
overcommitment, mean (SD)		14.2 (3.6)
Perceived quality of care, mean (SD)		1.9 (0.8)

^a^Only specialties reported by at least 5.0 % are listed

Table 2 shows the responses to the items of the quality of care measure. With the exception of omission of a diagnostic test or premature discharge of patients, each poor care behavior has ever been displayed by at least half of the sample. The most frequent behaviors, which physician engaged into on an at least monthly basis, were incomplete discussion of treatment options or responses to patient questions (reported by 18.4 %), discharge of patients to reduce the workload (14.3 %), and little attention for the social or personal impact of an illness (8.7 %).

**Table 2:** Perceived quality of care (individual items)^a^

Item	*n* ^b^	Response options, %				
		Never	Once	Several times per year	Monthly	Weekly
I found myself discharging patients to make the service “manageable” because the team was too busy.	379	59.6	7.9	18.2	9.0	5.3
I did not fully discuss treatment options or answer a patient’s question.	391	36.6	12.3	32.7	10.5	7.9
I made treatment or medication errors that were not due to a lack of knowledge or inexperience.	389	47.8	31.9	17.7	2.1	0.5
I did not perform a diagnostic test because of desire to discharge a patient.	377	69.0	8.8	17.8	2.9	1.6
I paid little attention to the social or personal impact of an illness on a patient.	389	47.3	12.9	31.1	5.9	2.8
I felt guilty about how I treated one of my patients from a humanitarian standpoint.	390	50.3	26.2	20.5	1.8	1.3

^a^Questionnaire items were developed by Shanafelt et al. [27]

^b^ *n* with complete data on the respective item

**Fig. 1:**
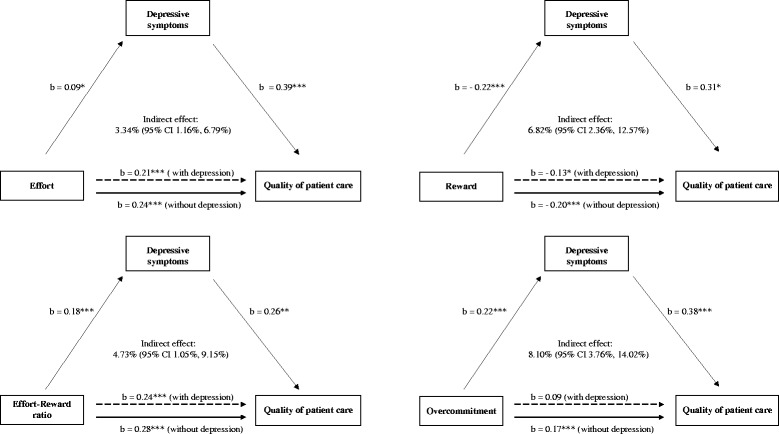
Mediation analyses of effort reward imbalance constructs (z scores) and quality of care (continuous variable), adjusted for age, sex, working environment and leadership position; * *p* <0.05, ** *p* < 0.01, *** *p* < 0.001

Figure 1 depicts the results from the multivariable linear regression mediation analyses. All ERI components showed a significant relationship with depressive symptoms, which in turn, were associated with poorer quality of care. In analyses unadjusted for depressive symptoms, increasing scores for effort, ERI ratio, and overcommitent were associated with poorer care while higher rewards were related to better patient care. Correction for depression partly attenuated all those associations. Notably, associations of effort, reward, and the ERI ratio with quality of care persisted after additional adjustment for depressive symptoms. By contrast, the overcommitment-care relationship was weak and was attenuated to non-significant levels after adjustment for depressive symptoms.

Table 3 shows results from the logistic regression analyses, which largely confirm the pattern of associations suggested by linear regression models: Effort, reward and the ERI ratio showed meaningful associations with poor care. These relationships were observed irrespectively of adjustment for depressive symptoms, which resulted in partial and weak attenuation of ORs. By contrast, overcommitment showed weak links with quality of care and adjustment of depression brought about a non-significant association of marginal magnitude.

**Table 3:** Association of effort-reward imbalance with perceived poor quality of care (logistic regression; secondary analyses)^a^

		Model I^b^		Model II^c^		Model III^d^	
		OR^e^	95 % CI^f^	OR	95 % CI	OR	95 % CI
Effort	Low	1	ref	1	ref	1	ref
	High	2.19	1.41, 3.41	2.31	1.42, 3.76	2.06	1.26, 3.39
	z-score	1.75	1.37, 2.22	1.83	1.40, 2.40	1.75	1.33, 2.31
Reward	Low	1	ref	1	ref	1	ref
	High	0.51	0.30,0.86	0.50	0.11,0.89	0.63	0.35,1.16
	z-score	0.65	0.51, 0.84	0.64	0.49, 0.84	0.71	0.53, 0.96
Effort-reward imbalance	Low	1	ref	1	ref	1	ref
	High	2.08	1.26,3.45	2.06	1.20,3.54	1.74	1.00,3.06
	z-score	1.87	1.43, 2.45	1.92	1.43, 2.57	1.79	1.32, 2.43
Overcommitment	Low	1	ref	1	ref	1	ref
	High	1.37	0.88,1.12	1.43	0.90,2.28	1.09	0.65,1.81
	z-score	1.32	1.06, 1.64	1.36	1.07, 1.72	1.18	0.91, 1.54

^a^Effort, reward, overcommitment, and quality of care were dichotomized based on the top tertile of the respective score distribution and the ERI ratio based on its established cut-off (i.e., high = ERI score >1; low = ERI score ≤ 1.0)

^b^Unadjusted

^c^Adjusted for age, sex, working environment and leadership position

^d^Additional adjustment for depressive symptoms

^e^OR = odds ratio

^f^CI = confidence interval

Finally, there was no evidence of an interaction between ERI and overcommitment in relation of quality of care. The *p*-value for the corresponding interaction term in the adjusted models equaled 0.84 (model without depression) and 0.98 (model with depression).

## Discussion

Our study examined relationships between the effort-reward-imbalance model of work stress and physicians’ perceptions of patient care quality. Addressing current research gaps, we specifically aimed a) to estimate the robustness of associations to statistical control for depressive symptoms, and b) to fully examine ERI model constructs as determinants. We observed that initial associations of effort, reward and the ERI ratio with quality of care were only partially explained by physicians’ depressive symptoms, i.e. those associations were of meaningful strength regardless of adjustment for physicians’ depressive symptoms. The link between overcommitment and quality of care was weak and attenuated to a non-significant relationship by adjustment for depressive symptoms. Further, overcommitment did not modify the association between the ERI ratio and quality of care.

### Comparison to the literature and potential explanations

Our main finding that decreasing quality of care is associated with higher work stress is consistent with prior work [9]. Findings from similar studies based on other well-established work stress models, beyond the ERI model, confirm that physicians’ adverse work conditions are inversely related with the quality of care they are able to provide [33–35]. Additionally, the current evidence supports links between work stress and various indicators of quality of care, that is, not only physician-perceived care [14, 15] or patients’ experiences [14, 33, 36]. Instead, these relationships expand to objectively assessed outcomes such as medical errors [37] or clinical outcomes among patients, for instance, hospital-associated infections [38] or the level of glycemic control in diabetic patients [34, 35].

The findings from our study can be compared well to the prior study by Klein et al. [15], which employed both the ERI model and partly similar quality of care indicators in German surgeons. Two particular indicators seem to conceptually overlap with the content covered by our quality of care instrument: these are, a marker of “psychosocial care” (i.e. quality of informing patients about the rationale for treatment, considering psychosocial aspects of patients’ illness, and showing empathy) and self-reported errors related to diagnosis and treatment. Klein et al. found a high ERI ratio (versus low ERI) to be associated with poorer psychosocial care (OR =1.38) and increased errors (OR = 1.24) [15]. The latter estimate did not reach statistical significance, however. Nevertheless, if synthesized with our observations, the current evidence suggests moderate associations of ERI and physician-perceived quality of care. Our study expands this prior work by Klein et al. [15] through separate consideration of effort and reward, both of which showed meaningful associations with quality of care in our study. Consistent with Klein et al. [15], our study suggests that any link between physicians’ overcommitment and quality of care is at most marginal. In addition, our findings did not support the notion that overcommitment may modify the association between the ERI ratio and physician-perceived quality of care.

In contrast to quantitative studies, qualitative studies are able to provide a more detailed understanding of the factors that physicians may themselves view as key contributors to poorer care. Although the number of such qualitative studies is limited, they suggest that work stress may primarily contribute to poorer care through physicians’ fatigue and tiredness (induced by high efforts). Based on their qualitative study in Scottish hospitals, Ross et al. [39] reported that physicians consistently identified high workload, time pressure and frequent interruptions as key causes for their medical errors. These work characteristics are well-captured by the effort component of the ERI model [17]. Another qualitative study in UK physicians revealed tiredness to be the major contributor to poor patient care while depression and anxiety were perceived as far less relevant [40]. These findings lend support to the tentative hypothesis that tiredness and mental workload may be the primary mediators linking high efforts (i.e., high work and patient load) to poor health care practices such as poor discharge practices and prescribing errors [39] as well as poor attention to patients during consultations. In our study, depressive symptoms were only marginally attenuating and thus only partially explaining associations between effort and quality of care, which is in line with prior reports [15]. Possibly depressive symptoms are not a suitable indicator of work-induced tiredness, which may be less severe and transient and is thus reduced by recovery from work in many physicians. High efforts may further contribute to poor care because physicians have too little time to re-attend to tasks they had to previously leave uncompleted, e.g., due to interruptions [14, 39]. Thus, even if physicians wanted to resume incomplete or correct insufficient care practices, high efforts may constrain their ability to make any timely corrections or improvements (e.g. due to a high workload time is lacking to return to a patient physicians assume to have mistreated). Another plausible explanation for our findings is that display of poor care practices represents a coping strategy among physicians: when facing high demands, physicians may strive to reduce their workload by engagement into behaviors such as premature discharge of patients, restriction of time for social interaction, and omission of diagnostic tests.

We further found that increasing rewards (i.e. perceptions of receiving a fair salary, high recognition, having high job security and good promotion prospects) were associated with better patient care irrespectively of physicians’ depressive symptoms. It has been suggested that in particular reward (rather than effort) is a strong determinant of employees’ job satisfaction [41, 42], which itself is associated with the perceived quality of care [43]. Alternatively, one may assume that physicians in well-designed work environments are capable to provide better-quality care and thus receive corresponding acknowledgment (e.g., by superiors and colleagues), which is a key characteristic of our reward measure. Due to the cross-sectional nature of our study we cannot rule out though that the potential direction of this association is reversed. Future studies are needed to confirm the suggested link between reward and patient care and to examine potential mediators of that association.

### Strengths and limitations

Beyond the above-mentioned contributions, our study has specific methodological strengths such as, first, measurement of psychosocial work conditions by an established work stress model [17], which allows for theory-based assessments and interventions. Second, we were able to include physicians from various medical specialties who are employed in different health care sectors. This raises confidence regarding the external validity and generalizability of our observations. Finally, we were able to control our estimates with a reliable measure for poor mental health (i.e. depressive symptoms), which may (partially) account for observed associations.

Despite these strengths, limitations of our study need to be mentioned. First, as highlighted above, our study was cross-sectional and does thus not allow inferring causality from the observed relationships. Second, although we provided documentation of the psychometric properties of our quality of care measure, we acknowledge that our outcome measure was applied for the first time in Germany. Since this instrument was drawn from an US-based study, further investigations into its validity and reliability in non-English speaking healthcare settings may be warranted. In addition, that instrument comprises one particular item, which may have limited suitability for physicians working in outpatient care (i.e. the item on discharge practices). Physicians in outpatient care may have referred to their past working experience in hospitals (i.e. during residency) when replying to that item. To explore the potential for information bias, we re-ran our primary analyses excluding that item and found similar results: e.g., the multivariable-adjusted b (including correction for depressive symptoms) for effort, reward, the ERI ratio and overcommitment in relation to quality of care were 0.19 (*p* < 0.0001), −0.12 (*p* = 0.03) 0.21 (*p* < 0.0001) and 0.08 (*p* = 0.08), respectively. A third limitation relates to our restricted sample size, which did not allow for stratification (e.g., by sex, health care sector, amount of working experience) to further explore associations and to identify risk groups. Fourthly, our study had a good response rate given the number of follow-ups and the duration of the follow-up period (72.5 %). Nevertheless, selection bias induced by differential non-response participation may be of concern. We therefore compared participants and non-participants of the 2014 assessment (*n* = 450 vs. *n* = 171) with regard to key characteristics (Table 1) reported at the baseline in 2004. These groups did not differ significantly in terms of age, sex distribution, and levels of depressive symptoms, effort, reward and overcommitment (data not shown). We are thus confident that any potential selection processes and subsequent bias is likely marginal. Finally, our and relevant prior studies [14, 15] have been carried out in German health care settings. Additional studies are thus needed to examine the transferability of our findings to other health care systems.

### Implications

If our findings are confirmed by prospective evidence, interventions are needed to promote physicians’ work life thereby improving quality of care. It has been demonstrated that the reduction of demands and efforts as well as the promotion of perceived rewards is feasible in hospital settings, e.g. through participatory intervention strategies [44, 45]. Further, an intervention study addressing interruptions among hospital physicians, which is conceptualized as a type of effort by the ERI model [17], showed that reduction of interruptions translated into better care as perceived by patients and physicians [14]. Potentially, simultaneously addressing multiple sources of efforts and rewards in future interventions studies may magnify these beneficial effects on the quality of care. Future studies based on longitudinal designs are needed to test the generalizability of our findings to other types of care indicators as well as their specific associations with physicians’ work life. These insights may inform the development of effective interventions to improve the work environment of physicians and the quality of care they provide.

## Conclusions

In summary, we found that high efforts and low rewards are associated with reports of poorer patient care among physicians. Depressive symptoms did not affect these associations. If corroborated by future prospective studies our findings suggest that, in terms of the ERI model, quality of patient care may be improved by reducing effort and increasing perceived rewards among physicians.

### Availability of data and materials

Request for the data used in this study should be send to Prof. Peter Angerer (email: Peter.Angerer@uni-duesseldorf.de).
